# The roles of exosomal immune checkpoint proteins in tumors

**DOI:** 10.1186/s40779-021-00350-3

**Published:** 2021-11-08

**Authors:** Cheng Xing, Heng Li, Rui-Juan Li, Le Yin, Hui-Fang Zhang, Zi-Neng Huang, Zhao Cheng, Ji Li, Zhi-Hua Wang, Hong-Ling Peng

**Affiliations:** 1 Department of Hematology, The Second Xiangya Hospital, Central South University, Changsha, 410011 China; 2 Institute of Molecular Hematology, Central South University, Changsha, 410011 China; 3 Hunan Key Laboratory of Tumor Models and Individualized Medicine, Changsha, 410011 China

**Keywords:** Exosomes, Tumor, Immune checkpoints

## Abstract

Targeting immune checkpoints has achieved great therapeutic effects in the treatment of early-stage tumors. However, most patients develop adaptive resistance to this therapy. The latest evidence demonstrates that tumor-derived exosomes may play a key role in systemic immune suppression and tumor progression. In this article, we highlight the role of exosomal immune checkpoint proteins in tumor immunity, with an emphasis on programmed death ligand 1 (PD-L1) and cytotoxic T lymphocyte-associated antigen 4 (CTLA-4), as well as emerging evidence on roles of T cell immunoglobulin-3 (TIM-3), arginase 1 (ARG1), and estrogen receptor binding fragment-associated antigen 9 (EBAG9) expressed by exosomes.

## Background

Immune checkpoints are signaling pathway molecules expressed by immune cells that regulate immune responses while maintaining self-tolerance and are regarded as the gatekeepers of immune responses [1]. Multiple immune checkpoints have been characterized in the past decades, and various studies have demonstrated their roles in tumor progression through enhancement of anti-tumor immune responses. Representative inhibitory immune checkpoints include cytotoxic T lymphocyte-associated antigen 4 (CTLA-4), programmed death ligand 1 (PD-L1), lymphocyte activation gene-3 (LAG-3), T cell immunoglobulin-3 (TIM-3), and the V-domain immunoglobulin-containing suppressor of T-cell activation (VISTA); examples of stimulating immune checkpoints include OX40 (CD134), 4-1BB (CD137), inducible costimulator (ICOS), etc. [2]. Among these markers, particular attention has been paid to CTLA-4 and PD-L1, with preliminary progress achieved with the use of their neutralizing antibodies for therapeutic intervention in the clinical setting. This mode of therapy has been accredited by the United States Food and Drug Administration (FDA) for clinical use [1, 2]. However, this treatment approach poses certain limitations in the clinical setting, including development of adaptive resistance in a majority of patients, unsatisfactory overall response rates [3, 4], and adverse reactions such as development of autoimmune symptoms [2, 5]. Encouraging results have been observed in some preclinical studies and/or clinical trials of other novel immune checkpoints [6, 7].

Expression of various immune checkpoint proteins has been detected in exosomes. Exosomes are extracellular vesicles (EVs) of 40–150 nm in diameter and are bioactive lipid bilayer nanovesicles secreted by almost all types of normal or tumor cells. Exosomes can carry various molecules (e.g., proteins, lipids, DNA, and RNA) that are mainly involved in intercellular signal communication [8–11]. However, there may be significant differences in exosomal activity among different cell types. Furthermore, exosomes can carry cell-type-specific proteins with specific fates and functions [10]. Exosomes secreted by the same cell contain distinct components, and the different numbers of exosomes can be secreted under different stimuli [8]. A large number of studies have shown that exosomes are involved in a variety of physiological and pathological processes [12, 13] and promote or inhibit the occurrence of diseases such as autoimmune diseases [14], kidney diseases [15], brain diseases [16, 17], bone diseases [18], and cardiac diseases [19]. Exosomes play a pivotal role in steps of tumor progression [20–22], including tumor cell proliferation [23, 24], angiogenesis [25, 26], and metastasis [27, 28]. Exosomes are an important part of the tumor microenvironment (TME) and can exert a predictive role in the state of the TME to a certain extent [29]. As important carriers of cell content exchange, exosomes have attracted widespread attention for their role in chronic lymphocytic leukemia (CLL) [30], ovarian cancer (OvCa) [26], pancreatic cancer [23, 31, 32], gastric cancer [27], esophageal cancer [33], colorectal cancer [34], liver cancer [35], and other types of cancer, in suppressing immune responses and regulating the TME [21, 22, 36, 37]. The cargo carried by exosomes has been proven to promote inflammation, angiogenesis, tumor growth, and metastasis [38]. For example, leukemia cell-derived exosomes can transform monocytes into “tumor-associated macrophages” and release inhibitory growth and anti-apoptotic factors beneficial for the expansion of leukemia cells [38]. The proteins of tumor-derived exosomes (TEXs) are delivered to endothelial cells through endocytosis, inducing angiogenesis and promoting tumor growth [25]. The biologically active proteins carried by exosomes can inhibit the cytotoxicity and regulate the expression of immune-related genes in T cells to promote tumor immune escape [39]. Extensive investigation of the components of exosomes has revealed the expression of various immune checkpoint proteins, such as PD-L1, CTLA-4, and TIM-3, in exosomes, especially in TEXs. An increasing number of researchers believe that immune checkpoint proteins in exosomes are involved in a novel mechanism that mediates tumor immune escape [40–43]. Accumulated evidence supports the hypothesis that these checkpoint molecules may act as new targets for cancer immunotherapy, with immune checkpoint blockade acting as a promising method for activating anti-tumor immunity [1, 7, 44]. Blocking the secretion of exosomes in addition to the immune checkpoints may further enhance the effectiveness of anti-tumor immune responses and offer new insights for tumor immunotherapy. Further, exosomes derived from normal cells and tumor cells are quite different in terms of number and composition, reflecting specificity to a certain extent, and are present in various body fluids [30, 38, 45, 46]. Thus, the detection of exosomes has potential value in early disease diagnosis and the prognostic evaluation of tumors [20, 47]. In this article, we reviewed the impact of various tumor-derived exosomal immune checkpoints on immune function in recent years, and summarized the important value of exosomes in clinical and scientific research.

## Functions of exosomal immune checkpoint proteins in cancer

**Fig. 1:**
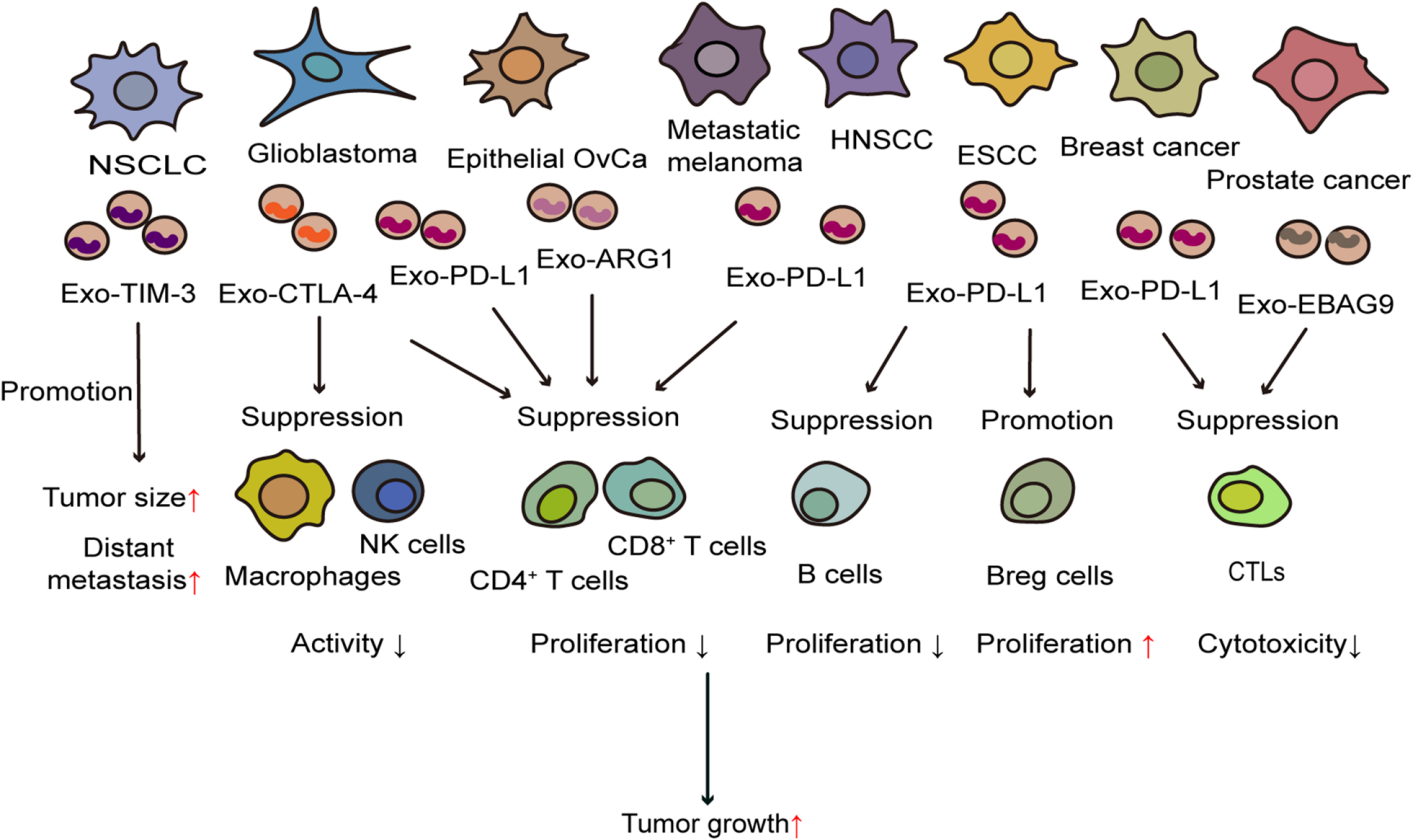
Exosomes carrying immune checkpoint proteins can inhibit immune cell function and promote tumor progression. Exo-TIM-3 derived from NSCLC cells positively correlates with larger tumor size and worse distant metastasis. Exo-CTLA-4 secreted by glioblastoma cells suppress the activation of immune cells including CD4^+^ T cells, CD8^+^ T cells, NK cells and macrophages. Glioblastoma-derived Exo-PD-L1 and epithelial OvCa-derived Exo-ARG1 both inhibit the proliferation of CD4^+^ T cells and CD8^+^ T cells. Exo-PD-L1 secreted by HNSCC cells or metastatic melanoma cells suppresses the function of CD8^+^ T cells. Besides, Exo-PD-L1 from ESCC cells inhibits the proliferation of B cells and induces an increase in PD-1^high^ Breg cells. The cytotoxicity of CTLs could be inhibited by breast cancer-derived Exo-PD-L1 and prostate cancer-derived Exo-EBAG9. ↑ Promotion; ↓ Inhibition; Exo-TIM-3 Exosomes containing T cell immunoglobulin-3; NSCLC Non-small-cell lung cancer; Exo-CTLA-4 Exosomes containing cytotoxic T lymphocyte-associated antigen 4; NK cells Natural killer cells; Exo-PD-L1 Exosomes containing programmed death ligand 1; OvCa Ovarian cancer; Exo-ARG1 Exosomes containing arginase 1; HNSCC Head and neck squamous cell carcinoma; ESCC Esophageal squamous cell carcinoma; CTLs Cytotoxic T lymphocytes; Exo-EBAG9, Exosomes containing estrogen receptor binding fragment-associated antigen 9

The immune system plays a vital role in tumor occurrence and development. Tumors can evade the immune system through various mechanisms. Increasing evidence shows that TEXs are carriers of immunosuppressive proteins [48]. TEXs carry cargo to regulate tumor progression by regulating the TME. The proteins and nucleic acids carried by exosomes are reflective of the cells of their origin. Many studies have found that exosomes carry immune checkpoint proteins, such as PD-L1, CTLA-4, and TIM-3 [49–53]. A substantial increase has been observed in the expression of immune checkpoint proteins in exosomes of tumor cells compared with those of healthy controls in several studies [43, 49, 54]. Furthermore, the level of immune checkpoints in exosomes exhibited a definite correlation with malignant parameters based on tumor characteristics, clinical stage, and lymphatic metastasis [43, 49, 52]. Many experiments have confirmed that exosomes expressing immune checkpoint proteins can promote tumor progression and metastasis [39, 51, 55]. However, blocking immune checkpoints could relieve the immune suppression induced by exosomes and inhibit tumor growth. We believe tumor immunosuppression mediated by exosomal immune checkpoint proteins may play an important role in immunotherapy resistance, as is supported by previous research. Herein, we summarize recent studies that have attempted to link immune checkpoint proteins carried by TEXs with their targets and describe their regulation in tumor immunity (Table 1, Fig. 1).

**Table 1:** Regulation of immune checkpoint proteins carried by TEXs in tumor immunity

Origin of exosomes	Immune checkpoint proteins carried by exosomes	Targeted cells	Effect	References
PC3, prostate cancer cell lines	PD-L1	Jurkat T cells	Suppress Jurkat T cells activation	[51]
TRAMP-C2, human prostate cancer-like cells	PD-L1	CD4^+^/CD8^+^ T cells	Suppress CD4^+^ and CD8^+^ T cells activation	[51]
Glioblastoma cells	PD-L1	CD4^+^ T cells, CD8^+^ T cells	Suppress T cell activation and proliferation	[56]
ESCC cells	PD-L1	B cells, Breg cells	Inhibit the proliferation of B cells; induce an increase in B10 and PD-1^high^ Breg cells; activate TLR4 and MAPK signaling pathways	[54]
Breast cancer cells	PD-L1	CTLs	Promote tumor growth	[55]
HNSCC cells	PD-L1	CD8^+^ T cells	Suppress T cell activation	[52]
Metastatic melanoma cells	PD-L1	CD8^+^ T cells	Suppress the function of CD8^+^ T cells; facilitate tumor growth	[57]
MEL624 cells, human melanoma cell lines	PD-L1	CD8^+^ T cells	Inhibit the proliferation, cytokine production and cytotoxicity of CD8^+^ T cells; reduce expression of Ki-67 and GzmB, inhibit the production of IFN-γ, IL-2, and TNF-α	[57]
B16-F10 cells, mouse melanoma cell lines	PD-L1	CD8^+^ T cells	Inhibit the proliferation and cytotoxicity of mouse splenic CD8^+^ T cells	[57]
Glioblastoma cells	CTLA-4	CD4^+^ T cells, CD8^+^ T cells, NK cells, macrophages	Inhibit the immune cell function	[48]
NSCLC cells	TIM-3	–	Positively correlate with larger tumor size, advanced stages and more distant metastasis	[49]
Epithelial OvCa cells	ARG1	CD4^+^ T cells, CD8^+^ T cells	Inhibit the proliferation of antigen-specific T cells; accelerate tumor progression	[43]
ID8-ARG1-V5 cells	ARG1	BMDCs	Inhibit CD8^+^ and CD4^+^ T cells proliferation	[43]
Prostate cancer cells	EBAG9	CTLs	Inhibit the cytotoxicity of T cells; negatively regulate tumor surveillance in host cells	[39]

*TEXs* tumor-derived exosomes, *PD-L1* programmed death ligand 1, *ESCC* esophageal squamous cell carcinoma, *Breg* regulatory B cell, *B10* interleukin‐10^+^ Bregs, *CTLs* cytotoxic T lymphocytes, *HNSCC* head and neck squamous cell carcinoma, *GzmB* granzyme B, *IFN-γ* interferon-γ, *IL-2* interleukin-2, *TNF-α* tumor necrosis factor-α, *CTLA-4* cytotoxic T lymphocyte-associated antigen 4, *NK cells* natural killer cells, *NSCLC* non-small-cell lung cancer, *TIM-3* T cell immunoglobulin-3, *OvCa* ovarian cancer, *ARG1* arginase 1, *ID8-ARG1-V5* murine ID8 OvCa model overexpressed V5-tagged murine ARG1, *BMDCs* marrow-derived dendritic cells, *EBAG9* estrogen receptor binding fragment-associated antigen 9

## Roles of exosomal PD-L1

PD-L1 is a type I transmembrane protein that binds to its receptor (PD-1) and inhibits the activation of T cells thus, maintaining immune homeostasis. High expression of PD-L1 is observed in tumor cells, which can protect these cells from T cell-mediated immune surveillance, via binding of PD-L1 to PD-1 expressed on activated T cells. Previous studies have shown that PD-L1 can be detected in most exosomes secreted by various types of tumor cells [52, 56–58]. The tumor immunosuppressive mechanism mediated by the interaction between exosomal PD-L1 secreted by tumor cells and PD-1 on activated immune cells has been confirmed in subsequent independent studies [57, 59, 60]. Blocking of PD-L1 with an antibody was used clinically to activate the anti-tumor immune response, leading to lasting remission in some cancer patients [61]. However, adaptive resistance occurred in most patients [61]. It is noteworthy that blocking exosomal PD-L1 using anti-PD-L1 antibody could add to an anti-tumor immune response and inhibit tumor growth more effectively [51]. Thus, exosomal PD-L1 is a novel therapeutic target to overcome the issue of resistance to antibodies observed in current methods.

### Functions of exosomal PD-L1 in tumor

In vitro experiments have proven that exogenous exosomal PD-L1 could rescue the growth of tumors that cannot secrete exosomes independently [51]. Conversely, studies have observed inhibited growth of wild-type tumor cells exposed to tumor cells deficient in exosomal PD-L1 [51]. For instance, glioblastoma has been shown to produce EVs carrying PD-L1, which directly bind to the PD-1 of T cells to suppress T cell activation and proliferation. Anti-PD-1 antibodies have been shown to significantly reverse the inhibitory effect of exosomes on T cell activation [56]. Exosomes from esophageal squamous cell carcinoma (ESCC) patients and ESCC cell lines inhibited the proliferation of B cells and induced an increase in interleukin‐10^+^ Bregs and PD‐1^high^ Bregs [54]. Meanwhile, research demonstrates that ESCC-derived exosomes could promote the expression of PD-1 and the secretion of IL-10 in recipient B cells, which may be related to the activation of TLR4 and MAPK signaling pathways [54]. In addition, breast cancer cells secrete tumor-derived microparticles containing high levels of PD-L1 after radiotherapy, which inhibits the activity of cytotoxic T lymphocytes (CTLs) and promotes tumor growth in vitro and in vivo [55]. In addition, blocking the PD-1/PD-L1 axis partially alleviated the TEX-mediated inhibition of CTL progression [55].

### Effect of exosomal PD-L1 on immunotherapy and biomarkers

PD-L1 carried by TEXs has the potential to become a biomarker for guiding treatment, evaluating drug resistance, and tracking prognosis (Table 2). Interferon-γ (IFN-γ) can upregulate PD-L1 on exosomes [57]. In patients with head and neck squamous cell carcinoma (HNSCC), exosomal PD-L1 levels are related to disease activity, the Union for International Cancer Control (UICC) staging, and lymph node status [52]. However, no clear correlation was found between the level of soluble PD-L1 (sPD-L1) and disease progression in patients with HNSCC [52]. Compared with normal controls, the level of PD-L1 on circulating exosomes in metastatic melanoma patients substantially increased, while the number of exosomes and total exosomal proteins differed slightly [57]. A previous study found that PD-L1 expression in tumor cells within lung squamous cell carcinoma was higher than that in adenocarcinoma cells [62]. Furthermore, in terms of its role in prognosis, pancreatic ductal adenocarcinoma (PDAC) patients who had exosomal PD-L1 stained positively with antibodies against PD-L1 experienced a shorter survival time after surgery [58]. In addition, in the case of melanoma and non-small cell lung cancer (NSCLC) [63], the level of PD-L1 in plasma-derived exosomes was substantially lower in patients with treatment response and higher in patients with disease progression, while no significant changes were observed in patients with stable disease (SD) [63]. Similar to previous studies, another study reported that high levels of circulating exosomal PD-L1 in patients with melanoma responded poorly to anti-PD-1 treatment [57]. Interestingly, the level of PD-L1 in peripheral blood exosomes and the re-invigoration of CD8^+^ T cells was meaningfully increased in patients who responded to initial anti-PD-1 treatment within 6 weeks of therapy [57]. This indicates that the PD-L1 levels of circulating exosomes before and after anti-PD-1 treatment might represent the different status of anti-tumor immunity [57]. If the level of PD-L1 in the peripheral blood exosomes of patients was beyond a breaking point before treatment, the anti-tumor activity of T cells was severely inhibited and could not be rescued. However, for the patients on anti-PD-1 treatment, CD8^+^ T cells that secreted IFN-γ were re-invigorated, and the levels of exosomal PD-L1 were upregulated by IFN-γ. Since the PD-L1/PD-1 interaction had been blocked by anti-PD-1 antibodies, the anti-tumor effect of T cells could not be inhibited. Potential reasons for the conflicting results of these two studies [57, 63] may include the inconsistent baseline levels of PD-L1 in patients before treatment or a lack of separation of exosomes derived from tumor cells or normal cells in the patients’ plasma. A large number of studies on various types of tumors have indicated that it is feasible to dynamically measure the expression of PD-L1 in plasma-derived exosomes, and circulating exosomal PD-L1 is expected to be a potential biomarker of disease progression as well as a prospective predictor of anti-PD-1 therapy [52, 56, 58]. In patients with melanoma, the use of therapeutic plasma exchange (TPE) has been reported to separate sPD-L1 and PD-L1-positive extracellular vesicles (evPD-L1) from plasma in vivo [50]. The efficacy of immunotherapy may be enhanced by using TPE to remove PD-L1 from exosomes in the peripheral circulation. Currently, anti-PD-1 antibodies are widely used for PD-1/PD-L1 blockade therapy, and anti-PD-L1 antibodies have shown clinical activity in some ongoing early clinical trials [64]. Whether the level of exosomal-PD-L1 is similarly affected by anti-PD-L1 remains to be confirmed.

**Table 2:** Application of PD-L1 carried by TEXs in evaluation drug resistance and tack prognosis

Cancer	Expression of PD-L1 carried by TEXs	Effect	Material	Sample	References
HNSCC	↑	Disease progression	PB	40 HNSCC patients	[52]
PDAC	+	Shorter postoperative survival time	PB	55 PDAC patients	[58]
Melanoma	↓	Respond to treatment	PB	18 melanoma patients	[63]
NSCLC	↓	Respond to treatment	PB	8 NSCLC patients	[63]
Metastatic melanoma	↑	Distinguish melanoma patients from healthy donors	PB	Patients with stage III to IV melanoma and healthy donors	[57]
Melanoma	↑	Fail to respond to the anti-PD-1 treatment	PB	Melanoma patients	[57]
Melanoma	↑	T cell re-invigoration	PB	Melanoma patients on pembrolizumab therapy within 6 weeks	[57]
Glioblastoma	↑	Larger glioblastoma tumor volume	PB	21 glioblastoma patients	[56]

“↑” Increased, “↓” Decreased, “+” Positive, *TEXs* tumor-derived exosomes, *PD-L1* programmed death ligand 1, *HNSCC* head and neck squamous cell carcinoma, *PB* peripheral blood, *PDAC* pancreatic ductal adenocarcinoma, *NSCLC* non-small-cell lung cancer, *anti-PD-1* anti-programmed death 1

### Effects of TEXs on PD-L1/PD-1 expression on immune cells

Apart from tumor cells, PD-L1 is also expressed on immune cells, such as dendritic cells (DCs), monocytes, and macrophages. TEXs can regulate the expression of PD-L1 on these immune cells and inhibit tumor immunity [65–69]. Exosomes derived from hepatocellular carcinoma (HCC) cells can upregulate the expression of PD-L1 on THP-1 cells or RAW264.7 cell-differentiated macrophages, while exosomes treated with melatonin can downregulate the expression of PD-L1, which is related to regulation of the STAT3 signaling pathway [65]. Similarly, exosomes secreted by glioblastoma-derived stem cells were found to induce monocytes to differentiate into an immunosuppressive M_2_ phenotype with upregulation of PD-L1; this process was also regulated by the STAT3 signal pathway [66]. Liu et al. [67] found that HCC cell-derived exosomes produced by endoplasmic reticulum stress that contain high levels of microRNA (miRNA/miR)-23a-3p could increase the expression of PD-L1 on macrophages through the PTEN/AKT pathway, resulting in the suppressed immune functions of T cells. Another study has confirmed a highly abundant RNA species in CLL-derived exosomes and identified that noncoding Y RNA hY4, which induces PD-L1 expression in monocytes and promotes the development of CLL [68]. Furthermore, exosomes from LLC Lewis lung carcinoma or 4T1 breast cancer cells could upregulate PD-L1 expression on DCs, which inhibits CD4^+^ T cell proliferation, and this immunosuppressive effect could be rescued by anti-PD-L1 antibodies [69]. It remains to be confirmed whether the high levels of PD-L1 on these immune cells are due to tumor-derived exosomal PD-L1. Interestingly, Qiu et al. [70] first found that activated T cells of triple-negative breast cancer (TNBC) patients could secret exosomal PD-1, but not PD-L1. Activated T cell-derived exosomal PD-1 could either induce the internalization of PD-L1 on the surface of tumor cells via endocytosis or neutralize the tumor cell-derived exosomal PD-L1, both of which could exert a preventive role in the interaction of PD-L1 with PD-1 on the T cell surface, rescue the activity of tumor-specific CTLs, and restore tumor surveillance. However, whether other immune cells secrete exosomal PD-1/PD-L1 and affect tumor progression is an issue worthy of further investigation and discussion.

## Functions of exosomal CTLA-4

CTLA-4 (CD152) is a type I transmembrane protein that is transiently expressed on the surface of T cells within 24–48 h after activation. CTLA-4 is an important negative regulator of T cell immune response. It forms a homodimer, competes with CD28, and has a higher affinity for CD80 (B7-1) and CD86 (B7-2). CTLA-4 can reduce CD80/86 expression on antigen-presenting cells (APCs) through trans-endocytosis to prevent CD28 costimulation [71].

A study found that IgG2a isotype anti-CTLA-4 antibodies had the most effective anti-tumor effects compared with other isotype antibodies in mouse tumor models [72]. Anti-CTLA-4 antibodies selectively mediated the reduction of regulatory T cells (Tregs) at tumor sites, inhibited the negative regulatory effects on tumors, and enhanced anti-tumor effects [72]. Glioblastoma-derived exosomes have been confirmed to carry a variety of immunosuppressive proteins including CTLA-4, which inhibit the immune function of CD8^+^ T cells, CD4^+^ T cells, natural killer (NK) cells, and macrophages [48]. For instance, the TEX and T cell-derived exosomes of the patients were serially monitored in a phase I clinical trial of HNSCC patients who received a combination of cetuximab, ipilimumab (a CTLA-4 antagonist), and radiation therapy. Expectedly, compared with patients who experienced recurrence within 2 years after treatment, CD3^+^ CTLA-4^+^ exosomes in disease-free patients declined significantly [73]. Additional studies are required to identify the roles and mechanisms of exosomal CTLA-4 in tumor immunotherapy in the future.

## Emerging immune checkpoints

The well-known immune checkpoints CTLA-4 and PD-1/PD-L1 play important roles in regulating the function of immune cells [44, 74]. Immune checkpoint inhibitors (ICIs), anti-CTLA-4, and anti-PD-1/PD-L1 monoclonal antibodies have been used as therapeutic agents clinically [7, 44]. As of the end of 2018, the FDA has approved up to seven types of ICIs for the standard treatment of 13 types of cancers, all of which are immune checkpoint blockers against PD-1/PD-L1 or CTLA-4 [75]. Unfortunately, anti-CTLA-4 or anti-PD-1/PD-L1 treatments have not been effective in many patients [4], with some patients exhibiting serious adverse reactions [5], such as neutropenia, autoimmune hemolytic anemia, and immune thrombocytopenia [76]. Many researchers have explored the therapeutic potential of other immune checkpoints; TIM-3, LAG-3, and T cell immunoglobulin and ITIM domains (TIGIT) are under exploration, with promising results obtained in mediating anti-tumor immunity [5, 6, 77].

### Exo-TIM-3

Transmembrane, immunoglobulin, and mucin (TIM)-3 belong to the T cell/TIM gene family, which is located on human chromosome 5q33.2. It can produce inhibitory signals expressed on T cells, DCs, macrophages, and mast cells [78, 79]. Galectin-9, the ligand of TIM-3, induces Th1 cell death and downregulates Th1 response. TIM-3/Galectin-9 suppresses tumor immunity by negatively regulating T cells. TIM-3 can induce immune tolerance and is associated with asthma and autoimmune diseases [77, 78]. Furthermore, and importantly, the expression of TIM-3 on the tumor-infiltrating lymphocytes (TILs) of glioma has been positively correlated with disease severity but negatively correlated with the Karnofsky Performance Status (KPS) score, indicating that TIM-3 is involved in the progression of glioma [80]. Research on patients with renal cell carcinoma (RCC) found that blocking the TIM-3 pathway could inhibit the activation of the TIM-3 pathway to restore the proliferation of CD4^+^ and CD8^+^ TILs and increase the production of IFN-γ [81]. The interaction of TIM-3 specifically expressed on tumor-associated dendritic cells (TADCs) and the nuclear protein high mobility group box 1 (HMGB1) could inhibit the activation of nucleic acid-mediated anti-tumor immune responses [79]. Meanwhile, simultaneous administration of anti-TIM-3 monoclonal antibody to tumor-bearing mice in conjunction with chemotherapy resulted in tumor regression [79]. Moreover, it has been confirmed that TIM-3/Galectin-9 signaling is a key pathway for tumor immune escape [79, 80]. TIM-3 is expected to be a new target for the treatment of malignancies. To date, anti-TIM-3 monoclonal antibodies have been used within clinical trials [6].

Few studies have reported on TIM-3 in exosomes, despite the existence of various studies based on tumor tissues or TILs to reveal the important role of TIM-3/Galectin-9 signaling in tumors. Gao et al. [49] reported, for the first time, the existence of TIM-3 in human peripheral circulating exosomes. In that study, the total exosomal protein (Exo-pro), exosomal TIM-3 (Exo-T), and exosomal Galectin-9 (Exo-G), isolated from the plasma of NSCLC patients, were related to several clinicopathological parameters [49]. Compared with healthy controls, plasma levels of Exo-pro, Exo-T, and Exo-G in NSCLC patients were statistically significantly higher and were positively correlated with tumor malignant parameters (including tumor size, progression, and metastasis) [49]. However, the high level of Exo-T was statistically significantly related to lymph node metastasis, while high Exo-G expression was not associated with lymph node metastasis [49]. Interestingly, elderly patients seemed to have higher Exo-T/G levels than younger patients [49]. Furthermore, the expression of Exo-T/G in the plasma of squamous cell carcinoma patients was statistically significantly higher than that in adenocarcinoma [49].

### Exo-ARG1

Arginase has two isoenzymes (ARG1 and ARG2) that hydrolyze L-arginine into L-ornithine and urea products. ARG1 is a cytoplasmic protein that is mainly expressed in the liver as an enzyme in the urea cycle, whereas ARG2 is expressed as a mitochondrial protein in the peripheral tissues of various mammals. Zhang et al. [82] revealed that serum exosomes (SExos) contain functional ARG1, which is elevated in diabetic *db/db* mice and diabetic patients. It can be taken up by endothelial cells to inhibit the production of nitrogen dioxide (NO) and damage endothelial function [82]. This study revealed the importance of SExos in regulating endothelial function and vascular homeostasis. Meanwhile, SExos-ARG-1 causes vascular dysfunction under different conditions, such as hypertension and hyperlipidemia, which deserves further study [82]. Tumor-derived EVs have also been reported to contain ARG1. ARG1^+^ EVs were found to be abundantly expressed in the plasma and ascites of patients with epithelial OvCa but were not detected in the ascites of patients with benign ovarian cysts [43]. The enzymatic activity of ARG1 in EVs from OvCa cell line supernatant or ascites of OvCa patients was higher than that of EVs isolated from benign cyst fluid, and research has demonstrated that ARG1^+^ EVs could inhibit CD4^+^ and CD8^+^ T cells in a dose-dependent manner in ovarian carcinoma [43]. ARG1^+^ EVs can be phagocytosed by DCs or directly inhibit the proliferation of T cells and inhibit the proliferation of antigen-specific T cells to accelerate tumor progression [43]. The arginase inhibitor OAT-1746 antagonizes its inhibitory effects [43]. Collectively, ARG1^+^ EVS is a novel mechanism of tumor-induced systemic T cell dysfunction. Whether this mechanism is applicable to other tumor types that express arginase remains to be explored. Thus, ARG1^+^ EVs may be potential therapeutic targets.

### Exo-estrogen receptor binding fragment-associated antigen 9 (EBAG9)

EBAG9 was originally identified as an estrogen-responsive gene in breast cancer cells. EBAG9 has been found to be related to the pathophysiology of a variety of cancers, such as HCC, pancreatic cancer, and RCC [83–87], and has likewise been found to promote tumor progression and metastasis by inhibiting the cytotoxicity of immune cells [88]. Miyazaki et al. [39] found that cancer-derived EVs contain EBAG9 protein, which cooperates with transmembrane 9 superfamily member 1 (TM9SF1) to promote epithelial-to-mesenchymal transition (EMT) of prostate cancer cells, inhibits the cytotoxicity of T cells, and negatively regulates tumor surveillance in host cells to promote tumor development. EBAG9 monoclonal antibodies can rescue immune suppression mediated by Exo-EBAG9 by restoring the cytotoxicity of T cells [39]. EBAG9 may serve as a new type of immune checkpoint, and immunotherapy based on EBAG9 may be used as an alternative treatment option for patients with EBAG9 overexpression in the late stage.

## Prospects and challenges

Exosomes carrying immune checkpoint proteins can inhibit immune cell function and promote tumor progression. At present, the majority of studies on exosomal immune checkpoints emphasize exosomal PD-L1. In general, the peripheral circulation of exosomal PD-L1 in tumor patients has greatly increased relative to that in healthy controls. The level of exosomal PD-L1 is related to disease activity, clinical stage, and lymph node metastasis. In addition, plasma exosomes of tumor patients are related to treatment response and affect the survival of patients after treatment. These findings strongly support the view that exosomal PD-L1 plays an important role in mediating tumor growth and metastasis. CTLA-4, another important immune checkpoint, also has an immunosuppressive function similar to that of PD-L1. With the continuous exploration of emerging immune checkpoints, TIM3, ARG1, and EBAG9 have been confirmed to exist in exosomes and may be involved in the regulation of tumor progression.

However, the exosomes isolated from the plasma of tumor patients used in the current experiment were a mixture of TEXs and non-TEXs. To further explore the role of TEXs in regulating systemic immunosuppression, it is necessary to better explore exosome purification methods to distinguish the respective roles of TEXs and non-TEXs. Resistance or failure of immune checkpoint blocking therapy may be mediated by exosomes carrying immune checkpoint proteins. Based on previous research results, targeting exosomal immune checkpoints is expected to revolutionize tumor immunotherapy, and we hypothesize that inhibiting the secretion of TEXs has the potential to work synergistically by blocking immune checkpoint therapy to exert anti-tumor effects. Further understanding of the molecular mechanism of exosome secretion as well as blocking the secretion pathway of TEXs will be an important breakthrough. Moreover, removal of TEXs through TPE has potential clinical value. In addition, it also deserves further research to elaborate the molecular mechanism of exosome-mediated cell communication, the blockage of which is also of great significance in enhancing tumor immunotherapy. Nevertheless, more in-depth studies are needed to verify whether adjuvant therapy with simultaneous suppression of exosomal secretion as immunotherapy can optimize current immunotherapy strategies.

## Conclusion

A great deal of evidence has accumulated focusing on the role of exosomes in regulating the function of immune cells. TEXs have been shown to promote tumor progression and metastasis. In this article, we have reviewed the role of several immune checkpoint proteins expressed in exosomes in regulating tumor immunity. PD-L1, CTLA-4, TIM3, ARG1, and EBAG9 have been confirmed to exist in exosomes and may be involved in the regulation of tumor progression. To explore more accurate and specific regulatory mechanisms, it is necessary to emphasize the separation of exosomal components and the confirmation of corresponding identities. Whether there are other immune checkpoints in the complex components of exosomes requires further validation. We believe that through the continuous efforts of more professional scholars, a major breakthrough will be achieved in the field of exosomal immune checkpoints in the near future.

## Data Availability

Not applicable.

## Abbreviations

APCs: Antigen-presenting cells
ARG1: Arginase 1
CLL: Chronic lymphocytic leukemia
CTLA-4: Cytotoxic T lymphocyte-associated antigen 4
CTLs: Cytotoxic T lymphocytes
DCs: Dendritic cells
EBAG9: Estrogen receptor binding fragment-associated antigen 9
EMT: Epithelial-to-mesenchymal transition
ESCC: Esophageal squamous cell carcinoma
evPD-L1: PD-L1-positive extracellular vesicles
EVs: Extracellular vesicles
Exo-G: Exosomal Galectin-9
Exo-pro: Exosomal protein
Exo-T: Exosomal TIM-3
FDA: Food and Drug Administration
HCC: Hepatocellular carcinoma
HMGB1: High mobility group box 1
HNSCC: Head and neck squamous cell carcinoma
ICIs: Immune checkpoint inhibitors
ICOS: Inducible costimulatory
IFN-γ: Interferon-γ
KPS: Karnofsky Performance Status
LAG-3: Lymphocyte activation gene-3
miRNA: MicroRNA
NK: Natural killer
NO: Nitrogen dioxide
NSCLC: Non-small cell lung cancer
OvCa: Ovarian cancer
PD-1: Programmed death 1
PDAC: Pancreatic ductal adenocarcinoma
PD-L1: Programmed death ligand 1
RCC: Renal cell carcinoma
SD: Stable disease
SExos: Serum exosomes
sPD-L1: Soluble PD-L1
TADCs: Tumor-associated dendritic cells
TEXs: Tumor-derived exosomes
TIGIT: T cell immunoglobulin and ITIM domains
TILs: Tumor-infiltrating lymphocytes
TIM-3: T cell immunoglobulin-3
TM9SF1: Transmembrane 9 superfamily member 1
TME: Tumor microenvironment
TNBC: Triple-negative breast cancer
TPE: Therapeutic plasma exchange
Tregs: Regulatory T cells
UICC: The Union for International Cancer Control
VISTA: The V-domain immunoglobulin-containing suppressor of T-cell activation
